# Neutrophil Phenotypes in Coronary Artery Disease

**DOI:** 10.3390/jcm9051602

**Published:** 2020-05-25

**Authors:** Patrick Maréchal, Julien Tridetti, Mai-Linh Nguyen, Odile Wéra, Zheshen Jiang, Maxime Gustin, Anne-Françoise Donneau, Cécile Oury, Patrizio Lancellotti

**Affiliations:** 1 Department of Cardiology, University of Liège Hospital, 4000 Liège, Belgium; pmarechal@chuliege.be (P.M.); ju.tridetti@gmail.com (J.T.); mlnguyentrung@gmail.com (M.-L.N.); 2 Laboratory of Cardiology, GIGA Cardiovascular Sciences, University of Liège, 4000 Liège, Belgium; odile.wera@chuliege.be (O.W.); zheshen.jiang@uliege.be (Z.J.); Maxime.Gustin@uliege.be (M.G.); 3 Biostatistics Unit, Department of Public Health, University of Liège, 4000 Liège, Belgium; afdonneau@uliege.be; 4 Gruppo Villa Maria Care and Research, Anthea Hospital, 70123 Bari, Italy

**Keywords:** acute coronary syndrome, inflammation, neutrophil, outcome

## Abstract

Clinical evidence indicates that innate immune cells may contribute to acute coronary syndrome (ACS). Our prospective study aimed at investigating the association of neutrophil phenotypes with ACS. 108 patients were categorized into chronic stable coronary artery disease (*n* = 37), unstable angina (UA) (*n* = 19), Non-ST-Elevation Myocardial Infarction (NSTEMI) (*n* = 25), and ST-Elevation Myocardial Infarction (STEMI) (*n* = 27). At the time of inclusion, blood neutrophil subpopulations were analysed by flow cytometry. Differential blood cell count and plasma levels of neutrophilic soluble markers were recorded at admission and, for half of patients, at six-month follow-up. STEMI and NSTEMI patients displayed higher neutrophil count and neutrophil-to-lymphocyte ratio than stable and UA patients (*p* < 0.0001), which normalized at six-month post-MI. Atypical low-density neutrophils were detected in the blood of the four patient groups. STEMI patients were characterized by elevated percentages of band cells compared to the other patients (*p* = 0.019). Multivariable logistic regression analysis revealed that plasma levels of total myeloperoxidase was associated with STEMI compared to stable (OR: 1.434; 95% CI: 1.119–1.837; *P* < 0.0001), UA (1.47; 1.146–1.886; *p* = 0.002), and NSTEMI (1.213; 1.1–1.134; *p* = 0.0001) patients, while increased neutrophil side scatter (SSC) signal intensity was associated with NSTEMI compared to stable patients (3.828; 1.033–14.184; *p* = 0.045). Hence, changes in neutrophil phenotype are concomitant to ACS.

## 1. Introduction

Increasing clinical evidence supports the existence of a link between low-grade systemic inflammation and cardiovascular (CV) risk [1]. Plasma level of high-sensitive C-reactive protein (hs-CRP) at the time of acute coronary syndrome (ACS) and its rise after the incident disease have been associated with a higher risk of major CV event [2]. CRP is produced by the liver in response to elevation of pro-inflammatory cytokines such as IL-6 and IL-1β, two well-recognized players in atherosclerosis progression. In the CANTOS trial [3], dampening inflammation by targeting the IL-1β pathway at the time of ACS lowered the risk of recurrent major CV events in patients who experienced a previous ACS. Hence, inflammation is currently considered as a modifiable CV risk factor. Among other inflammation-related risk factors, several studies pointed to the differential white blood cell count, with a particular focus on neutrophils, lymphocytes and monocytes. Neutrophil count by itself could predict both acute and chronic CVD [4,5]. Interestingly, an elevated neutrophil-to-lymphocyte ratio (NLR) has not only been associated with CVD but also with short-term adverse CVD outcomes, including mortality, coronary artery disease (CAD), stroke and heart failure [6]. Changes in NLR are subsequent to a modification of the balance between innate (neutrophils) and adaptive (lymphocytes) immunity. The association between elevated NLR and poor prognosis in cancer has also been linked to cancer-elicited release of immune suppressive neutrophils from the bone marrow [7,8,9]. In view of recent reports on the existence of common risk factors in CVD and cancer [10], it is therefore possible that CVD might similarly be associated with a mobilisation of neutrophils with altered phenotype, which might contribute to ACS risk. In chronic inflammatory diseases, as in cancer, peripheral blood neutrophils display diversity in their phenotype [8]. To date, neutrophil phenotypes in CAD patients and their association with ACS risk and outcome remain unexplored. Our study aimed at providing a first description of neutrophil phenotypes in prospectively recruited coronary artery disease patients. We investigated the association of neutrophil markers with ACS, their evolution after six months, as well as their capability to predict patient outcome during one-year follow-up.

## 2. Materials and Methods

### 2.1. Patients and Study Design

**Figure 1:**
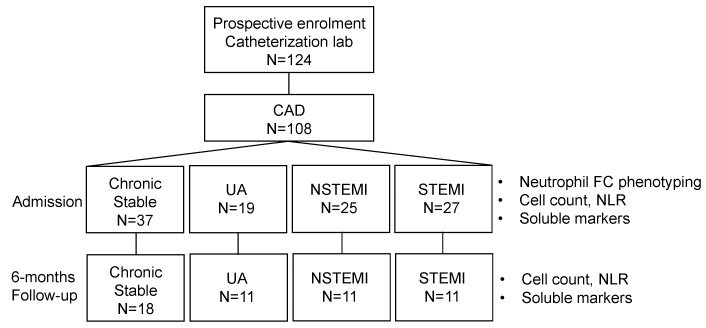
Study flow chart. CAD, coronary artery disease; FC, flow cytometry; NLR, neutrophil-to-lymphocyte ratio; NSTEMI, Non-ST-Elevation Myocardial Infarction; STEMI, Non-ST-Elevation Myocardial Infarction; UA, unstable angina.

A total of 124 patients admitted to our cardiac catheterization lab at University of Liège Hospital were prospectively recruited between March 2017 and October 2018 (Figure 1). Patients were classified as chronic stable coronary artery disease (angina symptoms/brief discomfort during exertion or physiological stress and with significant coronary lesions), unstable angina (UA, recent onset or worsening angina for up to two months, or resting angina, with or without electrocardiographic changes and significant coronary lesions), Non-ST-Elevation Myocardial Infarction (NSTEMI, troponin elevation above the 99-percentile associated with symptoms compatible with myocardial ischemia without significant elevation of the ST segment), and ST-Elevation Myocardial Infarction (STEMI, troponin elevation above the 99-percentile associated with symptoms consistent with myocardial ischemia with significant ST segment elevation). Patients who displayed angiographically healthy coronary arteries, myocardial infarction (MI) with nonobstructive coronary arteries (MINOCA), Takotsubo syndrome, spontaneous coronary artery dissection, myocarditis and pericarditis or those who did not sign the inform consent were excluded (16 patients out of 124). Patient demographic and clinical characteristics were recorded at admission. Biological variables were measured at admission and after > six months for a subset of patients (47%). The study was conducted according to the Declaration of Helsinki principles upon approval by the ethical committee of the University of Liège Hospital (B70720097066; 2009/205, accepted 11/24/2009).

### 2.2. Blood Samples, Sera and Platelet Poor Plasma (PPP) Preparation, Laboratory Measurements

Blood samples from patients were collected from radial or femoral site of arterial catheter and processed within 1 h. Blood samples were always obtained around midday to limit circadian oscillations of neutrophil phenotypes. Haematological parameters were determined in EDTA-anticoagulated blood on a Cell-Dyn 3700 (Abbott Laboratories). Platelet poor plasma was obtained after two consecutive centrifugations at 1750 g during 15 min at room temperature. Sera were obtained following one centrifugation at 1750 g during 15 min at RT. Plasma and sera were stored at −80 °C until analysis. IL-6 and S100A9 were measured on EDTA-plasma using a Luminex assay kit (R&D Systems, Minneapolis, MN, USA) according to the manufacturer’s instructions. Nucleosome levels were quantified on EDTA-plasma with the Cell Death Detection ELISA plus kit (Sigma-Aldrich, Overijse, Belgium). Myeloperoxidase quantity and activity were analysed on EDTA-plasma by a method adapted from Franck et al. [11] using a human myeloperoxidase (MPO) Quantikine ELISA kit (R&D Systems). hs-CRP, CK-MB (Abbott Laboratories, Wavre, Belgium) and high-sensitive troponin T (hs-TnT) (Roche, Machelen, Belgium) were measured in sera by routine immunoassays.

### 2.3. Normal and Low-Density Neutrophil Phenotyping

Low density neutrophils (LDNs) and normal density neutrophils (HDNs) were separated from EDTA-anticoagulated blood by density gradient centrifugation (Ficoll-Paque, GE Healthcare) under endotoxin-free conditions. The peripheral blood mononuclear cell (PBMC) fraction contains LDNs and the granulocyte fraction contains HDNs. LDNs and HDNs were phenotyped by flow cytometry using a panel of antibodies directed against CD45 (clone HI30, BD Biosciences), CD66b (clone G10F5, BioLegend), CD11b (clone ICRF44, BD Biosciences), CD16 (clone 3G8, BD Biosciences) and CD10 (clone HI10a, BioLegend) as previously described [12]. The gating strategy is shown in supplemental Figure S1. Data were acquired on a FACSVerse cytometer and analysed with the FACSuite software (BD Biosciences). Data included levels of CD11b, CD16 and CD10 expressed on the surface of CD66b positive LDNs and HDNs as well as percentages of neutrophils at different maturation stages in these two cell populations, e.g., segmented cells (CD10^+^CD16^+^CD11b^+/high^), band cells (CD10^−/low^CD16^int^CD11b^+^), metamyelocytes (CD10^−^CD16^low^CD11b^+^), myelocytes (CD10^−^CD16^−^CD11b^low^), and promyelocytes (CD10^−^CD16^−^CD11b^−^). Cytospins of LDNs and HDNs were performed after positive cell selection by magnetic bead cell sorting (MACS Miltenyi Biotech) using an anti-CD66b antibody (clone G10F5, BioLegend) according to the manufacturer’s instructions.

### 2.4. Statistics

Qualitative variables are summarized as numbers and percentages. Normality of the distribution of quantitative variables was tested by Shapiro-Wilk test. Quantitative variables with normal distribution were presented as mean ± SD, while median (interquartile range (p25–p75)) were reported for quantitative variables with skewed distribution. Qualitative characteristics of patient groups were compared by Chi-square or Fisher exact tests. ANOVA or Kruskal-Wallis tests were applied for quantitative variables as appropriate, with Tukey or DSCF post-hoc test, respectively. Spearman correlation analyses were performed to assess the association between LDN percentage and other quantitative variables. Multinomial logistic regression (MLR) was applied to model association between CAD categories and demographic, clinical and biological variables. Significant variables with *p* < 0.1 after univariate MLR analyses were considered into multivariable MLR analysis [13]. Results of the final MLR model were presented using odds ratios and corresponding 95% confidence interval. Cox proportional hazard models were used to investigate the occurrence of composite endpoint at 1-year follow-up (cardiovascular death, stroke, myocardial infarction or major bleeding) and to evaluate the prognostic value of changes in neutrophil markers from baseline to 6-month follow-up. Multivariable Cox model for composite endpoint at 1-year follow-up used the same variable selection method as for MLR. All hazard ratios (HR) were calculated with appropriate unit and corresponding 95% confidence interval. Concordance index (c-index) of the final model was calculated following Uno’s method and is presented as c-index with interquartile range (IQR). All tests were performed 2-sided and *p* < 0.05 was considered significant except when specified. Statistical analyses were performed using SAS 9.4 (SAS Institute, Tervuren, Belgium).

## 3. Results

### 3.1. Patient Characteristics, Inflammatory and Conventional Neutrophil Markers 

**Figure 2:**
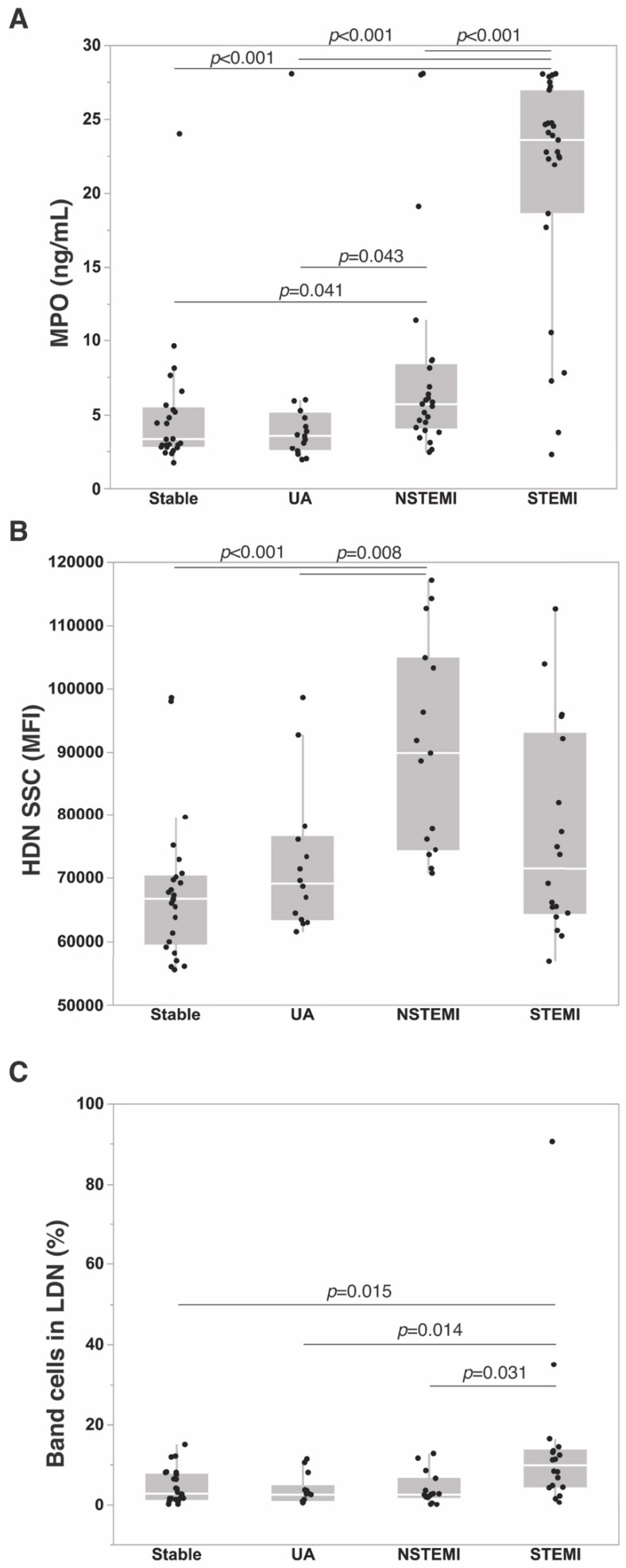
Neutrophil markers according to CAD category. (**A**) Plasma MPO levels. (**B**) Side scatter (SSC) signal intensity of high-density neutrophils (HDN) as determined by flow cytometry on blood granulocytic fraction. (**C**) Percentage of band cells in low-density neutrophils (LDN) isolated from peripheral blood mononuclear fraction. Data are presented using Tukey outlier box plots with box limits representing IQR and median in the middle, whiskers’ length are equal to 1.5 times of IQR.

A total of 108 patients were included: 37 (34%) patients had chronic stable coronary artery disease (stable), 19 (18%) UA, 25 (23%) NSTEMI, and 27 (25%) STEMI. Patient demographic and clinical characteristics according to diagnosis are depicted in Table 1. Patients from the four categories did not differ in terms of age, sex and conventional CVD risk factors. However, differences were observed in regard to aspirin (*p* = 0.0005) and lipid-lowering therapy (*p* = 0.001). Triglyceride levels were more elevated in STEMI patients than in UA (*p* = 0.009) and NSTEMI (*p* = 0.006). Regarding systemic inflammatory markers, the four patient groups displayed different levels of IL-6 (*p* = 0.003) (Table 2). As expected, differences in differential white blood cell counts were observed, mostly related to changes in neutrophil count. NSTEMI and STEMI patients had higher neutrophil counts than stable and UA patients (*p* < 0.0001; NSTEMI vs. UA *p* = 0.012). Among ACS, NSTEMI patients showed higher monocyte count than stable patients (*p* < 0.0001). In contrast, lymphocyte counts did not differ between patient categories. However, changes in NLR likely reflected the increase of neutrophil count. Plasma levels of S100A9, total and active MPO, and of nucleosomes, well-known circulating markers of neutrophil activation and neutrophil extracellular trap (NET) release that have previously been associated with CVD risk and ACS [14,15,16,17], were higher in STEMI patients than in stable (S100A9: *p* = 0.013; total MPO: *p* < 0.0001; active MPO: *p* < 0.0001; nucleosomes: *p* = 0.032) and UA (S100A9: *p* = 0.018; total MPO: *p* < 0.0001; active MPO: *p* < 0.0001; nucleosomes: *p* = 0.007) patients (Figure 2A). Despite similar increase of neutrophil count in NSTEMI and STEMI patients, the levels of these neutrophil markers were not significantly more elevated in NSTEMI patients than in stable and UA, suggesting that neutrophil phenotype, in addition to absolute cell numbers, differed between STEMI and other ACS conditions. Accordingly, total and active MPO levels were more elevated in STEMI than in NSTEMI patients (*p* < 0.0001) (Figure 2A). 

### 3.2. Neutrophil Phenotypes in ACS

**Figure 3:**
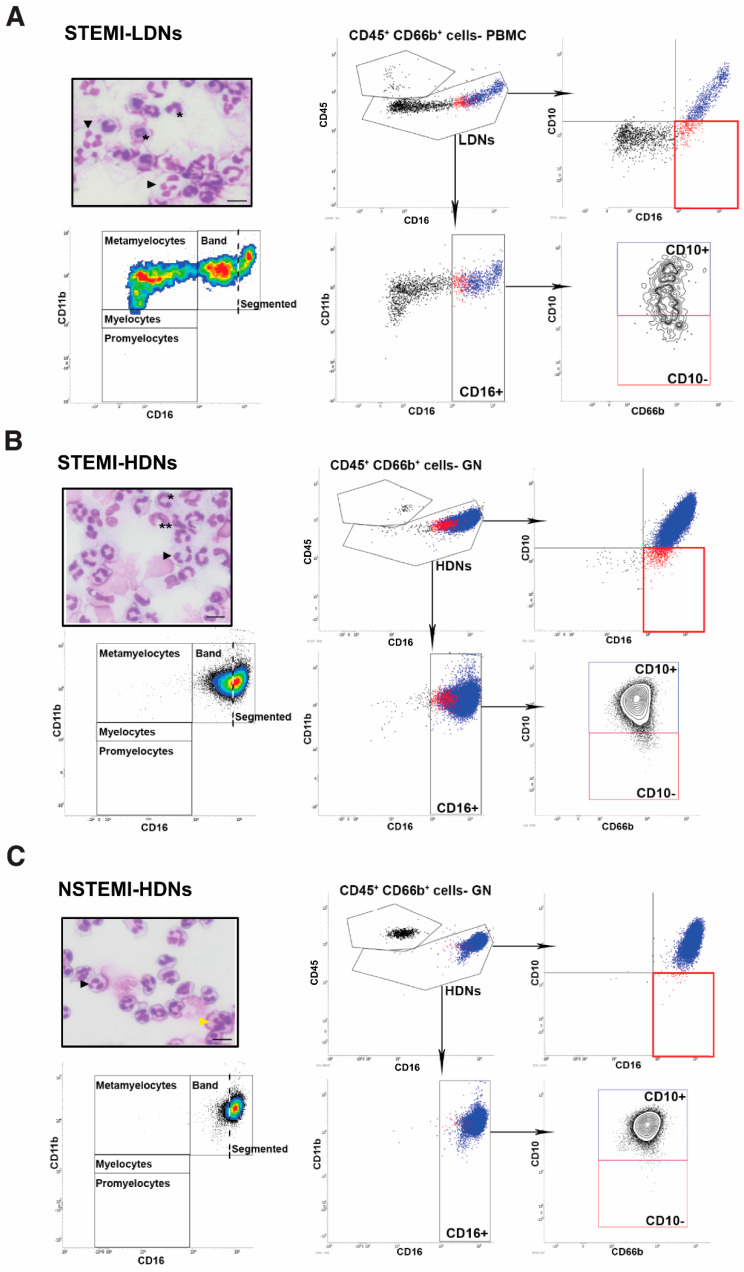
Neutrophil phenotype in the blood of NSTEMI and STEMI patients. (**A**) CD66b^+^ low density neutrophils (LDNs) from peripheral blood mononuclear cell (PBMC) fraction of STEMI patient blood. (**B**) and (**C**). CD66b^+^ high density neutrophils (HDNs) from the granulocytic (GN) fraction of STEMI and NSTEMI patient blood. Representative flow cytometry plots are shown depicting our phenotyping strategy (see Methods section for details). The red frame corresponds to band cells, represented in red on dot plots. Upper left panels represent cytospin images of sorted CD66b^+^ cells. Segmented cells (black arrowheads), larger segmented cells (yellow arrowhead), band cells (*) myelocytes (**). Bars represent 10 µm.

A detailed phenotypic analysis of neutrophils was performed for the four patient groups. We detected low percentages of LDNs in the peripheral blood mononuclear cells (PBMC) fraction isolated from patient blood, which were not different between patient groups (stable: 0.86 [0.36–1.8]; UA: 0.88 [0.39–1.77]; NSTEMI: 0.95 [0.38–2.1]; STEMI: 1.53 [0.57–6.69], *p* = 0.272). LDNs represented a heterogeneous cell population comprising variable proportions of myelocytes, band cells and segmented cells (Table S1). LDNs displayed features of immature cells, e.g., they were larger and expressed lower levels of CD11b, CD10 and CD16 than HDNs (Figure S2). The LDN percentage showed a modest correlation with the NLR (r = 0.32, *p* = 0.01), while it was not influenced by any other patients’ characteristics. Interestingly, STEMI patients were characterized by elevated percentages of band cells (CD10^−/low^CD16^int^CD11b^+^) in LDNs compared to the three other patient groups (*p* = 0.007) (Figure 2C, Figure 3A, and Table 3). Few band cells were detected in HDN fraction, with again higher percentages in the blood from STEMI patients than in stable (*p* = 0.047) and UA (*p* = 0.006) patients (Figure 2B–D and Table 1). HDNs from NSTEMI patients displayed higher complexity than those from stable (*p* = 0.003) and UA (*p* = 0.031) patients, as determined by SSC (side scatter) signal intensity (Table 1) (Figure 2B). There were no significant differences in CD11b, CD16 or CD10 expression levels on the surface of HDNs or LDNs from the four patient groups. Altogether, these data indicate that the increase of neutrophil count in ACS likely reflects elevated numbers of circulating neutrophils of high density. Distinct phenotypic neutrophil features were identified in STEMI and NSTEMI ACS. Elevated numbers of band cells were detected in the blood from STEMI patients, while NSTEMI patients were characterized by the presence of high-complexity HDNs. 

### 3.3. Neutrophil Markers in ACS

The association of these neutrophil markers with the four patient groups was assessed. After adjustment with confounding variables, i.e., age, gender, daily low-dose aspirin, and lipid-lowering therapy, plasma levels of total MPO (*p* < 0.0001), HDN SSC (*p* = 0.026) and hs-cTnT (*p* = 0.015) were independently associated with the four patient groups (Table 4). Plasma levels of total MPO were associated with STEMI compared to stable (OR per 1-ng/mL increase, 1.434; 95% CI, 1.119–1.837; *p* < 0.0001), UA (OR per 1-ng/mL increase, 1.47; 95% CI, 1.146–1.886; *p* = 0.002), and NSTEMI (OR per 1-ng/mL increase, 1.213; 95% CI, 1.1–1.1338; *p* = 0.0001) patients. Increased SSC intensity was associated with NSTEMI compared to stable (OR per 10000-MFI increase, 3.828; 95% CI, 1.033–14.184; *p* = 0.045).

Total MPO levels correlated with neutrophil count (*r* = 0.43, *p* = 0.0003) and levels of nucleosomes (*r* = 0.53, *p* < 0.0001). A moderate correlation was observed with S100A9 levels (*r* = 0.38, *p* = 0.0003), NLR (r = 0.30, *p* = 0.006), hs-TnT (*r* = 0.34, *p* = 0.002) and CK-MB (*r* = 0.24, *p* = 0.022). MPO levels were not influenced by any other patient characteristics or medication. HDN SSC showed a strong positive significant correlation with HDN FSC (*r* = 0.72, *p* < 0.0001) only. 

### 3.4. Evolution of Neutrophil Markers during Follow-Up

To assess time-dependent changes in neutrophil markers and to determine whether changes concomitant to myocardial injury were maintained at distance of ACS, some of these markers were measured after six months post-admission. Data could be obtained for 51 patients from the four categories (18 stable, 11 UA, 11 STEMI, 11 NSTEMI). Baseline characteristics of patients from this subgroup are shown in supplementary Table S2. Although neutrophil count (*p* < 0.0001), NLR (*p* = 0.0004) and MPO levels (*p* = 0.0009) and activity (*p* = 0.0002) differed between ACS and stable or UA patients at admission, these markers no longer differed between these patient groups at follow-up (Table S3). Likewise, stable patients who had a previous myocardial infarction (11 patients out of 37) did not differ in any neutrophil markers, i.e., NLR, MPO levels, LDN and band cell percentages, and HDN SSC (Table S4). These data suggest that mobilization of neutrophils harbouring a specific phenotype likely occurs at the time of myocardial infarction, which globally resolves over time. 

## 4. Discussion

Despite advances in understanding atherosclerotic processes, progression of CAD to acute events remains poorly understood and hardly possible to predict. Several studies indicate that neutrophils are involved in plaque progression [18,19,20,21], and preclinical data in mice suggest that low-grade systemic inflammation could promote atherosclerosis progression through neutrophil reprogramming [22]. Our study provides first clinical evidence that phenotypic modifications of circulating neutrophils occur around the time of MI. Moreover, our data suggest that these neutrophil phenotypes normalize with time. In addition, differences in neutrophil phenotypes were observed between STEMI and NSTEMI patients. 

### 4.1. Neutrophil Phenotypes in ACS

In CVD, it has been hypothesized that the NLR could reflect the release of immune suppressor cells (e.g., granulocytic myeloid suppressor cells, G-MDSC) from the bone marrow. In cancer and in various chronic diseases, G-MDSC can be isolated from the LDN neutrophil subset [23,24]. We identified variable percentages of LDN in the PBMC fraction of CAD patients’ blood, irrespective of disease stage. We showed too that neutrophils with altered phenotype circulate in the blood of STEMI and NSTEMI patients. We identified immature band cells in HDN and LDN from STEMI patient blood, while HDNs with high complexity were observed in the blood from NSTEMI patients. Circulating neutrophils from NSTEMI patients may therefore have distinct functionality and granular content. Band cells are neutrophils at intermediary stage of maturation, characterized by a curved nucleus. The appearance of these immature cells in the blood results from signals to the bone marrow for increased neutrophil production, as in the case of infection or inflammation. In STEMI, this probably indicates a response to severe myocardial damage with a high demand for neutrophils. Our observation that the LDN percentage moderately correlates with NLR could suggest an important biological role of these cells that might be independent of inflammation and conventional CV risk factors. In chronic inflammatory diseases such as lupus erythematous or psoriasis, low-density granulocytes that are more prone to produce NETs are thought to contribute to higher CVD risk [25,26]. For instance, the frequency of these cells has been associated with non-calcified coronary plaque burden in patients with psoriasis [27]. Whether LDNs from CAD patients possess immune suppressive or pro-inflammatory function or whether they are more susceptible to release NETs remains to be determined. 

### 4.2. Soluble Neutrophil Markers in ACS

We found elevated levels of soluble neutrophil activation markers, including MPO, S100A9 and nucleosomes in STEMI but not in NSTEMI patients, and this despite similar rise in neutrophil count and NLR. Total MPO levels appeared as a strong independent marker of STEMI. These distinct features between NSTEMI and STEMI might reflect different pathophysiological mechanisms leading to these ACS entities, which could not be picked up based on neutrophil count or NLR only. Noteworthy, it has recently been recognized that beside differences in ECG and troponin levels, plaque composition as well as mechanisms of atherogenesis influence CAD patient outcome [28,29,30]. MPO, stored in neutrophil azurophilic granules, is released upon neutrophil activation. Increased circulating levels of this protein have already been shown to predict the outcome of patients with ACS [15,16]. MPO would also be a marker of plaque vulnerability [16]. Interestingly, it has been reported that higher circulating MPO levels were found in ACS patients with culprit plaque erosion than in those with culprit plaque rupture [31]. It is noteworthy that in agreement with the study by Ferrante et al. [31], we did not find any correlation between MPO levels and markers of systemic inflammation, CRP or IL-6, while correlations were observed with other neutrophil-derived markers, like S100A9 and nucleosomes. Hence, neutrophil markers are likely to carry additional information compared to classical inflammatory markers since they may underlie biological mechanisms leading to ACS. 

### 4.3. Neutrophil Dynamics after ACS

In the study by Kim et al. [6], the shorter median time from ACS improved the strength of the association of NLR with a subsequent adverse event. Therefore, it has been suggested that the NLR would increase over time in CVD patients and reach a maximum around the time of event. In our study, in a subset of patients from each category, we could evaluate differential blood cell count, soluble neutrophil markers and inflammation at six-months post-MI. We showed that neutrophil count, NLR and soluble neutrophil markers returned to levels of stable patients during the follow-up period, therefore confirming that these markers reach a maximum around the time of ACS.

### 4.4. Clinical Implications

Our study highlights potential roles for neutrophil phenotypes as novel biomarkers in CAD. Advances in the cellular biology of atherosclerosis and its pathogenesis have led to major advances in its treatment. We provide first evidence that neutrophils could be a key player in the disease progression to ACS through changes in their phenotype and secretory profiles. Targeting pro-inflammatory or immune regulatory neutrophils (specific neutrophil subsets) and/or their downstream mediators that promote adverse effects (e.g., NET, pro-inflammatory cytokines, reactive nitrogen/oxygen species, neutrophil serine proteases, danger-associated molecular pattern molecules) may thus represent new therapeutic opportunities. Deoxyribonuclease (DNAse), an enzyme that breaks down NET, has been shown in a clinical study of STEMI patients to accelerate lysis of coronary thrombi [32]. Colchicine, an anti-inflammatory agent, has been shown to acutely suppress caspase-1 activity and inhibit inflammasome related cytokines in monocytes from ACS patients [33]. The use of colchicine in patients with recent myocardial infarction was also effective in reducing CV events [34]. Serine proteases liberated from activated neutrophils, e.g., cathepsin G, elastase, proteinase-3, are potent regulators of at least six IL-1 cytokine family members, including IL-1β [35]. One might consider inhibiting these extracellular proteases since this strategy will not affect neutrophil antimicrobial capability [35]. Neutrophils may indeed represent a major source of IL-1β in ACS. In cancer, IL-1β and S100A9 have been involved in the mobilization of pro-tumoral immature neutrophils with immune suppressive functions [36,37]. These molecules might also contribute to the production of immune suppressive neutrophils at the time of ACS. Neutrophils might also contribute to pathological oxidative stress, thereby altering the regulation of coronary blood flow by cardiac metabolism as seen in diabetes mellitus [38,39]. Hence, our study may pave the way toward new therapeutic avenues in ACS.

### 4.5. Limitations

The number of patients examined may be perceived as low. Nevertheless, our study of neutrophil profiles is unique, as it required good logistics coordination, involving both the laboratory and the clinics. Neutrophils need to be processed in a highly standardized manner within one-hour after blood collection. Blood samples were only obtained during working days. The neutrophil profile during follow-up was obtained in only half of the patients. Given the distinct function of each neutrophil subpopulation, it is likely that each subset participates in various pathophysiological mechanisms of CAD. The relationship between the neutrophil phenotypes and neutrophil function has not been studied. The relationship between neutrophil phenotype and scores of CAD severity has not been analysed. In addition, our study does not bring out a causal link between the neutrophilic profile and the destabilization of the coronary disease. It must be considered hypothesis-generating, even if the secretory and morphological dynamics of neutrophils are suggestive. Future larger studies evaluating the role of neutrophils in ACS onset and outcome are needed. 

## 5. Conclusions

Our study indicates that, in patients with CAD, changes in neutrophil phenotypes occur concomitantly to ACS. These changes may reflect different acute complications of CAD and reveal new biological processes leading to ACS.

## Figures and Tables

**Table 1:** Patient characteristics according to coronary artery disease (CAD) category.

	Stable *n* = 37	UA *n* = 19	NSTEMI *n* = 25	STEMI *n* = 27	*p*
Age (yrs)	69 ± 9	67 ± 11	63 ± 12	64 ± 10	0.081
Male gender, *n* (%)	25 (67.6)	17 (89.5)	19 (76)	20 (74.1)	0.356
Smoking, *n* (%)	24 (64.9)	12 (63.2)	15 (60)	22 (81.5)	0.343
Body mass index	27.8 (25.3–31.0)	28.7 (24.6–34.3)	26.8 (25.4–29.7)	26.9 (24.2–31.0)	0.616
Hypertension, *n* (%)	29 (78.4)	16 (84.2)	14 (56)	18 (66.7)	0.133
Hypercholesterolemia, *n* (%)	25 (67.6)	13 (68.4)	13 (52)	13 (48.1)	0.302
Diabetes, *n* (%)	13 (35.1)	8 (42.1)	5 (20)	8 (29.6)	0.425
Chronic renal failure, *n* (%)	5 (13.5)	3 (15.8)	1 (4)	4 (14.8)	0.530
Chronic inflammatory disease, *n* (%)	7 (18.9)	2 (10.5)	0 (0)	4 (14.8)	0.107
Active cancer, *n* (%)	0 (0)	2 (10.5)	2 (8)	4 (14.8)	0.069
History of DVT, *n* (%)	2 (5.4)	1 (5.3)	0 (0)	2 (7.4)	0.669
History of stroke, *n* (%)	4 (10.8)	2 (10.5)	0 (0)	0 (0)	0.086
History of MI, *n* (%)	11 (29.7)	4 (21.1)	1 (4)	7 (25.9)	0.095
History of CABG, *n* (%)	6 (16.2)	2 (10.5)	3 (12)	1 (3.7)	0.475
History of PCI, *n* (%)	12 (32.4)	10 (52.6)	5 (20)	8 (29.6)	0.145
Aspirin, *n* (%)	31 (83.8)	15 (78.9)	9 (36)	15 (55.6)	0.0005
DAPT, *n* (%)	5 (13.5)	1 (5.3)	3 (12)	3 (11.1)	0.886
Anticoagulant, *n* (%)	1 (2.7)	1 (5.3)	0 (0)	1 (3.7)	0.878
Lipid-lowering drug, *n* (%)	28 (75.7)	14 (73.7)	8 (32)	12 (44.4)	0.001
hs-cTnT (ng/L)	13 (8–23)	16 (10–20)	689 (303–1304) ^a,b^	293 (19–1438) ^a,b^	<0.0001
CK-MB (μg/L)	2.80 (2.00–4.50)	2.50 (1.80–3.50)	22.50 (14.80-60.00) ^a,b^	4.94 (2.50–47.56) ^a,b^	<0.0001
Creatinine (mg/dL)	1.08 (0.90–1.30)	1.00 (0.88–1.23)	0.94 (0.81–1.09)	0.95 (0.86–1.13)	0.207
Total cholesterol (mg/dL)	155 (138–181)	142 (126–208)	170 (143–211)	181 (142–195)	0.299
LDL (mg/dL)	87 (68–106)	78 (72–127)	109 (91–137)	111 (73–143)	0.052
HDL (mg/dL)	44 (36–53)	35 (29–45)	39 (34–48)	39 (34–49)	0.162
Triglycerides (mg/dL)	113 (88–180)	139 (100–197)	136 (101–165)	82 (62–114) ^b,c^	0.003
Apo A-I (g/dL)	1.32 (1.11–1.48)	1.14 (1.03–1.40)	1.29 (1.14–1.44)	1.21 (1.08–1.40)	0.497
Apo B (g/dL)	0.77 (0.61–0.90)	0.79 (0.74–1.05)	0.95 (0.85–1.08)	0.91 (0.70–1.07)	0.081
Lipoprotein (a) (nmol/L)	15 (8–70)	35 (10–118)	33 (10–135)	25 (9–116)	0.727
PCSK9 (pg/mL)	45514 (29768–85118)	55279 (23275–74669)	58512 (27008–78619)	76762 (33957–127092)	0.307

Abbreviations: Apo A-I, apolipoprotein A-I; Apo B, apolipoprotein B; CABG, coronary artery bypass grafting; CK-MB, creatine kinase-myocardial band; DAPT, dual antiplatelet therapy; DVT, deep venous thrombosis; HDL, high density lipoprotein; hs-cTnT, high sensitivity cardiac troponin T; LDL, low density lipoprotein; MI, myocardial infarction; PCI, percutaneous coronary intervention. Continuous data are presented as median (IQR). ^a^ indicates *p* < 0.05 vs. stable; ^b^ *p* < 0.05 vs. UA; ^c^ *p* < 0.05 vs. NSTEMI; ^d^ *p* < 0.05 vs. STEMI.

**Table 2:** Haematological and inflammatory parameters according to patient category.

	Stable *n* = 37	UA *n* = 19	NSTEMI *n* = 25	STEMI *n* = 27	*p*
Lymphocyte count (1000/μL)	1.41 (1.06–1.77)	1.82 (0.95–2.24)	1.79 (1.39–2.20)	1.54 (1.20-2.13)	0.185
Monocyte count (1000/μL)	0.46 (0.33–0.58)	0.53 (0.39–0.76)	0.73 (0.59–0.92) ^a^	0.64 (0.46-0.86)	<0.0001
Neutrophil count (1000/μL)	3.0 (2.1–4.5)	3.9 (2.5–5.2)	5.8 (4.6–8.3) ^a,b^	7.4 (6.4-9.7) ^a,b^	<0.0001
Eosinophil count (1000/μL)	0.12 (0.06–0.25)	0.12 (0.05–0.24)	0.06 (0.04–0.13)	0.07 (0.04-0.13)	0.094
Basophil count (1000/μL)	0.05 (0.04–0.08)	0.06 (0.04–0.07)	0.06 (0.04–0.08)	0.06 (0.04–0.07)	0.98
Haematocrit (%)	42 (41–47)	44 (41–48)	45 (42–48)	42 (38–47)	0.346
Platelet count (1000/μL)	255 ± 71	243 ± 63	247 ± 81	279 ± 72	0.309
Mean platelet volume (fL)	7.8 (7.4–8.3)	7.8 (7.6–8.8)	7.8 (7.1–8.3)	7.6 (7.0–8.5)	0.455
NLR	2.3 (1.8–3.2)	2.2 (1.7–3.1)	3.7 (2.3–5.3) ^a^	4.9 (2.7–7.5) ^a,b^	<0.0001
PLR	157 (141–244)	138 (112–239)	144 (119–170)	174 (122–209)	0.212
hs-CRP (mg/L)	2.93 (0.84–6.87)	1.26 (0.58–4.51)	6.09 (2.62–19.55)	2.51 (0.68–13.22)	0.082
IL-6 (pg/mL)	1.7 (0.4–3.4)	0.7 (0.2–1.5)	2.8 (0.9–13.0)	3.0 (0.8–12.3)	0.033
S100A9 (pg/mL)	213 (142–399)	250 (126–361)	273 (213–466)	431 (292–621) ^a,b^	0.008
Active MPO (ng/mL)	1.5 (1.2–3.2)	1.4 (1.1–1.9)	2.0 (1.5–3.2)	8.4 (4.9–13.2) ^a,b,c^	<0.0001
Total MPO (ng/mL)	4.1 (2.9–7.2)	3.7 (2.6–5.5)	5.7 (4.1–8.1)	23.6 (18.6–27.0) ^a,b,c^	<0.0001
Nucleosomes (AU)	0.04 (0.02–0.11)	0.03 (0.02–0.06)	0.06 (0.04–0.15)	0.09 (0.05–0.22) ^a,b^	0.006

Abbreviations: hs-CRP, high-sensitivity C-reactive protein; MPO, myeloperoxidase; NLR, neutrophil-to-lymphocyte ratio; PLR, platelet-to-lymphocyte ratio. Data are presented as mean±SD or median (IQR). ^a^ indicates *p* < 0.05 vs. stable; ^b^ *p* < 0.05 vs. UA; ^c^ *p* < 0.05 vs. NSTEMI; ^d^ *p* < 0.05 vs. STEMI.

**Table 3:** Neutrophil phenotype according to patient category.

	Stable (*n* = 37)	UA (*n* = 19)	NSTEMI (*n* = 25)	STEMI (*n* = 27)	*p*
HDN					
SSC	67,704 (61,896–74,737)	68,632 (63,191–76,788)	83,690 (72,344–99,563) ^a,b^	72,985 (64,586–92,739)	0.034
FSC	100,264 (90,863–111,559)	98,243 (92,717–108,761)	119,603 (104,467–130,478)	105,721 (90,881–120,638)	0.108
CD11b (MFI)	12,108 ± 4070	12,236 ± 4527	13,798 ± 4300	14,150 ± 3541	0.215
CD10 (MFI)	12,254 ± 3501	12,840 ± 3353	12,170 ± 3931	11,756 ± 4358	0.463
CD16 (MFI)	94,082 (83,307–137,384)	97,458 (74,226–113,011)	82,009 (65,544–102,078)	83,194 (65,209–108,398)	0.344
Band cells (%)	0.03 (0.01–0.08)	0.01 (0.00–0.06)	0.02 (0.01–0.09)	0.11 (0.03–0.36) ^a,b^	0.019
LDN					
% in PBMC	0.86 (0.36–1.80)	0.88 (0.39–1.77)	0.95 (0.38–2.10)	1.53 (0.57–6.69)	0.272
Band cells (%)	2.5 (1.2–6.8)	2.7 (1.0–6.5)	2.7 (1.8–7.3)	9.5 (4.0–13.7) ^a,b,c^	0.007

Abbreviations: HDN, normal density neutrophil; LDN, low density neutrophil; MFI, median intensity of fluorescence; PBMC, peripheral blood mononuclear cells; SSC, side scatter; FSC, forward scatter. Data are presented as mean ± SD or median (IQR). ^a^ indicates *p* < 0.05 vs. stable; ^b^ *p* < 0.05 vs. UA; ^c^ *p* < 0.05 vs. NSTEMI; ^d^ *p* < 0.05 vs. STEMI.

**Table 4:** Multivariable multinomial logistic regression model for CAD category *.

Variable	Comparison	Unit	Odds Ratio (95% CI)	*p*
hs-cTnT (ng/L)	NSTEMI vs. Stable	10	1.062 (1.020–1.105)	*0.003*
	STEMI vs. Stable	10	1.061 (1.019–1.104)	*0.004*
	Unstable vs. Stable	10	0.953 (0.824–1.103)	0.288
	NSTEMI vs. Unstable	10	1.114 (0.961–1.291)	0.152
	STEMI vs. Unstable	10	1.113 (0.960-1.290)	0.156
	NSTEMI vs. STEMI	10	0.999 (0.994–1.004)	0.722
Total MPO (ng/mL)	NSTEMI vs. Stable	1	1.182 (0.919–1.519)	0.193
	STEMI vs. Stable	1	1.434 (1.119–1.837)	*<0.0001*
	Unstable vs. Stable	1	0.975 (0.857–1.11)	0.703
	NSTEMI vs. Unstable	1	1.212 (0.942–1.559)	0.135
	STEMI vs. Unstable	1	1.47 (1.146–1.886)	*0.002*
	STEMI vs. NSTEMI	1	1.213 (1.100–1.338)	*0.0001*
HDN SSC	NSTEMI vs. Stable	10000	3.828 (1.033–14.184)	*0.045*
	STEMI vs. Stable	10000	3.029 (0.899–10.202)	0.074
	Unstable vs. Stable	10000	1.101 (0.603–2.007)	0.755
	NSTEMI vs. Unstable	10000	3.478 (0.990–12.217)	0.052
	STEMI vs. Unstable	10000	2.752 (0.843–8.977)	0.094
	STEMI vs. NSTEMI	10000	0.791 (0.423–1.478)	0.462

* Adjusted for age, sex, daily low-dose aspirin, lipid-lowering therapy. Abbreviations: HDN, normal density neutrophil; hs-cTnT, high-sensitive cardiac troponin T; MPO, myeloperoxidase; SSC, side scatter. Comparisons with *p* values < 0.05 are shown in italics.

## Supplementary Materials

The following are available online at https://www.mdpi.com/2077-0383/9/5/1602/s1, Figure S1: Gating strategy for NDN and LDN phenotypic analysis by flow cytometry, Figure S2: Comparison between HDNs and LDNs from CAD patients, Table S1: Percentages of mature and immature neutrophils according to CAD category, Table S2: Characteristics of patient subgroup according to CAD category, Table S3: Hematological and inflammatory parameters according to patient category at admission and at 6-month follow-up, Table S4: Hematological, inflammatory and neutrophil markers according to previous MI.
