# Supplementary material for: Neutrophil Phenotypes in Coronary Artery Disease
Source: J Clin Med. 2020 May 25;9(5):1602. doi: 10.3390/jcm9051602 (PMC7290445; doi:10.3390/jcm9051602)
Supplement: Supplementary file 1 [file jcm-09-01602-s001.pdf]

Marechal et al.

SUPPLEMENTARY MATERIALS

Figures

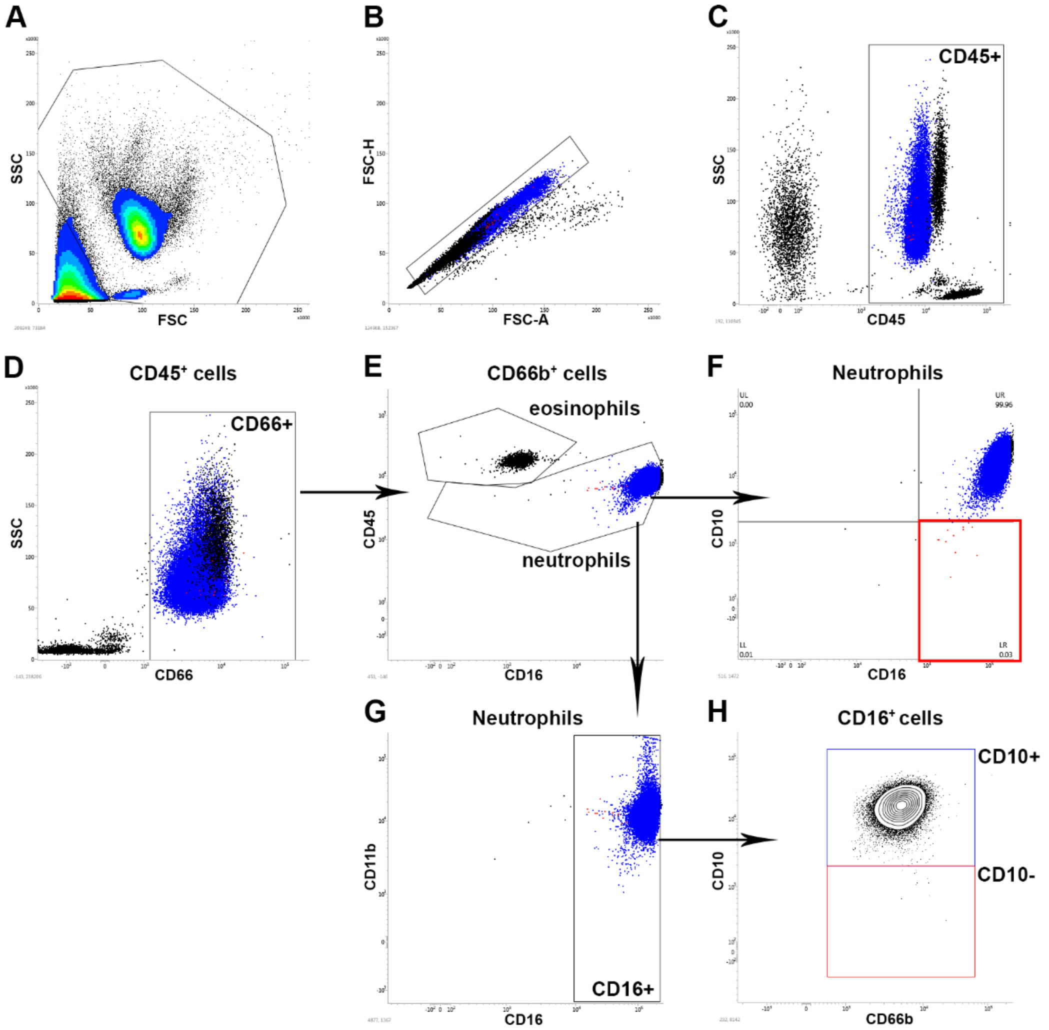

Figure S1. Gating strategy for NDN and LDN phenotypic analysis by flow cytometry. Representative plots of granulocytic fraction from chronic stable coronary artery disease patients (Ficoll gradient). (A) All events in forward scatter (FSC)-side scatter (SSC) plot. (B) Exclusion of doublets and cell aggregates. (C) Selection of $CD45^+$ cells (white blood cells). (D) $CD66b^+$ cells are selected among $CD45^+$ cells. (E) Selection of $CD45^+CD66b^+CD16^+$ neutrophils and exclusion of $CD45^{high}CD66b^+CD16^{low/-}$ eosinophils. (F) Plot of CD10 and CD16 expression on neutrophil surface. The red box depicts a $CD10^{-/low}CD16^{int}CD11b^+$ neutrophil subpopulation corresponding to immature band cells. (G) Plot of CD11b and CD16 expression on neutrophil surface. (H) $CD16^{+/high}$ neutrophils according to $CD10^+$ and $CD10^-$ phenotypes.

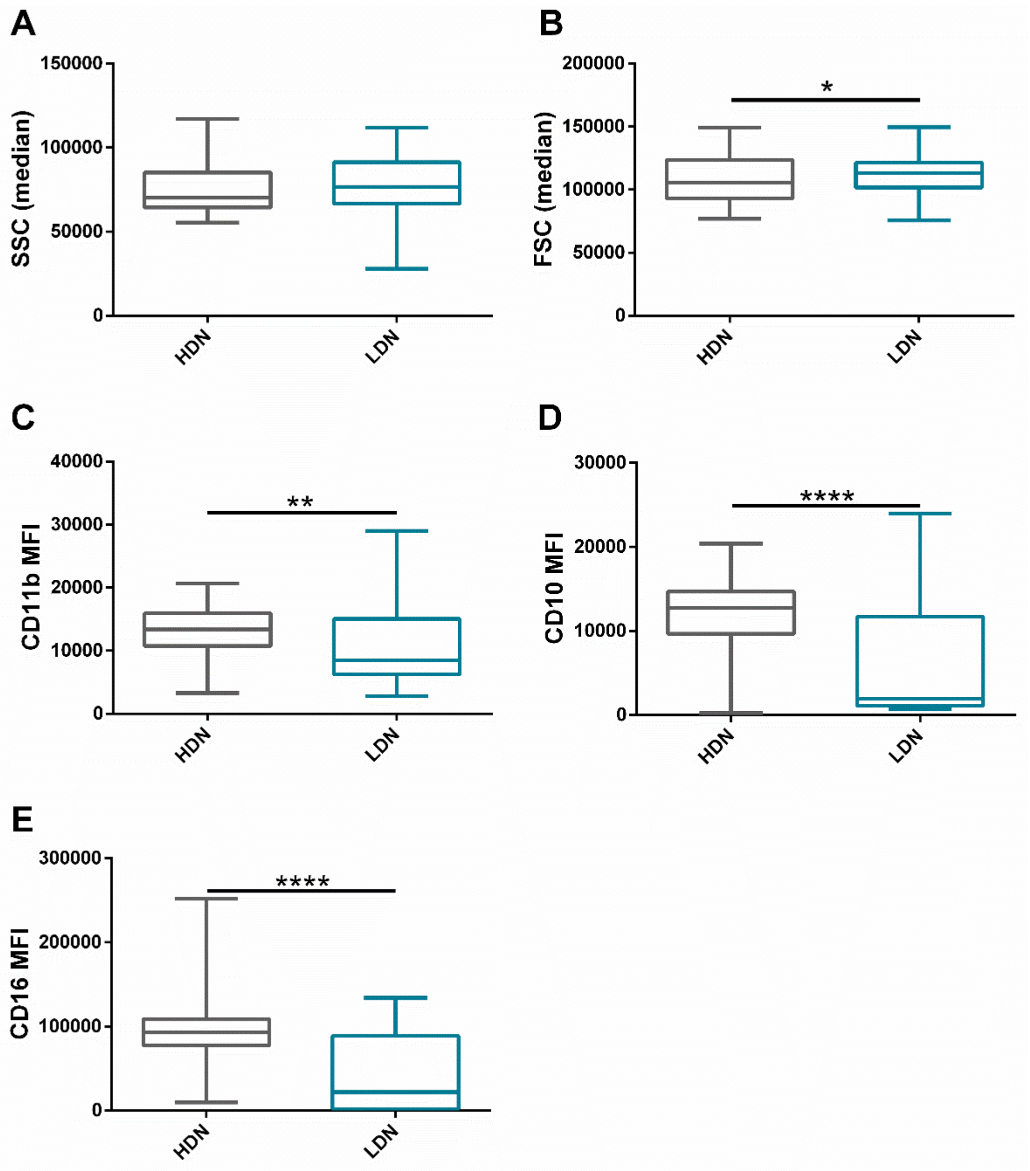

Figure S2. Comparison between HDNs and LDNs from CAD patients. Flow cytometry analysis of HDN and LDN from granulocytic and PBMC fraction of patient blood. Data represent medians of fluorescence intensity of indicated markers with minimum and maximum range. *$p<0.05$; **$p<0.01$; ****$p<0.0001$ (Mann-Whitney test).

Tables

Table S1. Percentages of mature and immature cells in HDN and LDN fractions according to CAD category

HDN	Stable	UA	NSTEMI	STEMI	P-value
Segmented cells (%)	99.98(99.9-100)	100 (99.9-100)	100 (99.9-100)	99.84 (99.6-100)	0.03
Myelocytes/metamyelocytes (%)	0 (0-0)	0 (0-0)	0 (0-0)	0.01 (0-0.02)	0.42
Band cells (%)	0.03 (0.01-0.08)	0.01 (0-0.06)	0.02 (0.01-0.09)	0.11 (0.03-0.36)[a,b]	0.019
LDN					
Segmented cells (%)	60.4 (43.3-86.3)	52.6 (9.7-89.4)	35.7 (13.1-82.2)	34.5 (13.3-72.3)	0.59
Myelocytes/metamyelocytes (%)	29.6 (10-44.2)	43.2 (6.2-79)	50.5 (4.9-80.1)	44.7 (11.7-75.1)	0.48
Band cells (%)	2.5 (1.2-6.8)	2.7 (1.0-6.5)	2.7 (1.8-7.3)	9.5 (4.0-13.7)[a,b,c]	0.007

HDN, normal density neutrophil; LDN, low-density neutrophil.
Data are presented as median (IQR). [a] indicates $P<0.05$ vs. stable; [b] $P<0.05$ vs. UA; [c] $P<0.05$ vs. NSTEMI

Table S2. Characteristics of patient subgroup according to CAD category

	All groups	Stable n=18	UA n=11	NSTEMI n=11	STEMI n=11	*P*
Age (yrs)	67 ± 9	71 ± 8	67 ± 10	62 ± 10	64 ± 7	0.053
Male gender, n (%)	39 (77%)	13 (72%)	10 (91%)	8 (73%)	8 (73%)	0.732
Smoking, n (%)	33 (65%)	12 (67%)	5 (46%)	8 (73%)	8 (73%)	0.565
Hypertension, n (%)	37 (73%)	15 (83%)	10 (91%)	5 (46%)	7 (64%)	0.074
Hypercholesterolemia	37 (73%)	14 (78%)	8 (73%)	7 (64%)	8 (73%)	0.906
Diabetes	19 (37%)	6 (33%)	7 (64%)	3 (27%)	3 (27%)	0.254
Chronic inflammatory diseases	6 (12%)	3 (17%)	2 (18%)	0 (0%)	1 (9%)	0.670
Chronic renal failure	4 (8%)	1 (6%)	1 (9%)	0 (0%)	2 (18%)	0.682
Cancer	4 (8%)	0 (0%)	1 (9%)	1 (9%)	2 (18%)	0.212
Previous deep vein thrombosis	2 (4%)	0 (0%)	1 (9%)	0 (0%)	1 (9%)	0.414
Previous myocardial infarction	10 (20%)	3 (17%)	2 (18%)	1 (9%)	4 (36%)	0.503
Previous stroke	3 (6%)	3 (17%)	0 (0%)	0 (0%)	0 (0%)	0.237
Previous CABG	7 (14%)	4 (22%)	1 (9%)	1 (9%)	1 (9%)	0.727
Percutaneous PCI	17 (33%)	4 (22%)	6 (55%)	2 (18%)	5 (46%)	0.186
Aspirin	37 (73%)	16 (89%)	9 (82%)	4 (36%)	8 (73%)	0.021
DAPT	6 (12%)	1 (6%)	1 (9%)	2 (18%)	2 (18%)	0.770
Anticoagulant	1 (2%)	0 (0%)	0 (0%)	0 (0%)	1 (9%)	0.647
Lipid-lowering therapy	34 (67%)	13 (72%)	8 (73%)	5 (46%)	8 (73%)	0.464
hs-cTnT	22 (12-120)	16 (9-33)	17 (7-25)	689 (258-1168)	31 (12-1317)	0.002
CK-MB	3.8 (2.2-14.8)	3.6 (2.4-5.0)	2.9 (1.6-3.9)	28.9 (14.8-60.0)	3.1 (1.6-119.4)	0.024
Creatinine	1.0 (0.9-1.2)	1.1 (0.9-1.3)	1.1 (0.9-1.2)	1.0 (0.9-1.1)	0.9 (0.9-1.2)	0.577
Total cholesterol	157 (133-197)	151 (136-176)	145 (130-196)	166 (142-238)	157 (116-197)	0.618

LDL	96 (72-135)	90 (68-112)	78 (74-98)	109 (84-175)	111 (72-143)	0.502
HDL	39 (34-50)	44 (34-57)	36 (29-44)	39 (35-48)	38 (34-52)	0.594
Triglycerides	106 (83-163)	96 (71-120)	135 (96-197)	138 (101-165)	85 (63-108)	0.049
Apo A-I	1.3 (1.1-1.4)	1.3 (1.2-1.4)	1.1 (1.0-1.5)	1.3 (1.2-1.4)	1.2 (1.1-1.4)	0.630
Apo B	0.9 (0.8-1.1)	0.8 (0.6-0.9)	0.8 (0.8-1.0)	0.9 (0.8-1.3)	0.9 (0.7-1.1)	0.597
Lp(a)	17 (7-83)	10 (7-83)	14 (9-72)	14 (7-203)	57 (18-80)	0.574
PCSK9 (pg/mL)	47756 (30549-84091)	42570 (30549-70386)	55279 (23275-61177)	39481 (20208-87812)	75768 (34250-135009)	0.309

Abbreviations: Apo A-I, apolipoprotein A-I; Apo B, apolipoprotein B; CABG, coronary artery bypass grafting; CK-MB, creatine kinase-myocardial band; DAPT, dual antiplatelet therapy; DVT, deep venous thrombosis; HDL, high density lipoprotein; hs-cTnT, high sensitivity cardiac troponin T; LDL, low density lipoprotein; MI, myocardial infarction; PCI, percutaneous coronary intervention. Continuous data are presented as median (IQR).

Table S3. Hematological and inflammatory parameters according to patient category at admission and at 6-month follow-up

	All groups	Stable n=18	UA n=11	NSTEMI n=11	STEMI n=11	*P*
**Baseline**						
Neutrophil count (1000/µL)	4.0 (2.7-7.7)	2.7 (1.7-3.9)	2.9 (2.3-5.2)	4.5 (3.8-8.5) [a]	8.9 (7.1-11.6) [a,b]	<0.0001
Lymphocyte count (1000/µL)	1.4 (1.0-1.8)	1.2 (0.9-1.6)	1.3 (0.7-2.2)	1.8 (1.4-2.0)	1.4 (1.2-1.8)	0.221
Monocytes count (1000/µL)	0.5 (0.3-0.7)	0.4 (0.3-0.5)	0.5 (0.2-0.8)	0.7 (0.6-1.0)[a]	0.6 (0.5-0.8)[a]	0.004
Basophil count (1000/µL)	0.056 (0.037-0.074)	0.051 (0.028-0.080)	0.058 (0.029-0.074)	0.056 (0.032-0.071)	0.057 (0.040-0.069)	0.980
Eosinophil count (1000/µL)	0.064 (0.034-0.181)	0.125 (0.047-0.253)	0.064 (0.033-0.237)	0.047 (0.034-0.092)	0.040 (0.018-0.071)	0.133
Hematocrit (%)	44.3 ± 4.8	44.2 ± 4.8	44.2 ± 4.1	44.5 ± 5.7	44.4 ± 5.4	0.999
NLR	3.1 (2.1-5.3)	2.3 (1.9-3.1)	2.2 (1.6-4.2)	3.8 (2.5-4.4)	6.8 (5.3-8.4) [a,b]	0.0004
Platelet count (1000/µL)	254 (214-301)	273 (209-313)	241 (205-320)	241 (224-247)	289 (214-302)	0.539
PLR	176 (140-280)	188 (154-347)	178 (119-285)	128 (123-156)	197 (169-239)	0.131
Mean platelet volume (fL)	7.8 (7.4-8.9)	7.9 (7.4-9.0)	7.7 (7.5-8.5)	8.0 (7.5-8.4)	7.7 (7.0-9.0)	0.927
IL-6 (pg/mL)	1.1 (0.2-3.4)	1.3 (0.0-3.4)	0.7 (0.3-1.5)	1.9 (0.5-14.3)	0.8 (0.2-3.1)	0.652
MPO activity (ng/mL)	2.0 (1.3-6.3)	1.3 (1.1-1.5)	1.3 (1.1-1.5)	2.0 (1.5-2.5)	9.1 (6.2-14.3)	0.0002
MPO (ng/mL)	4.8 (2.9-23.9)	2.9 (2.8-4.4)	3.5 (2.7-4.8)	4.8 (3.1-6.0)	24.6 (22.4-27.9)	0.0009

Nucleosomes (AU)	0.036 (0.026-0.072)	0.027 (0.019-0.060)	0.035 (0.033-0.063)	0.038 (0.023-0.048)	0.060 (0.034-0.089)	0.177
S100A9 (pg/mL)	233 (124-404)	159 (120-282)	224 (126-361)	244 (90-448)	405 (204-593)	0.102
**6-month follow-up**						
Neutrophil count (1000/µL)	3.6 (3.0-4.8)	3.9 (3.3-4.6)	3.3 (2.9-5.0)	3.5 (3.0-4.6)	3.9 (2.8-5.7)	0.838
Lymphocyte count (1000/µL)	1.7 (1.5-2.1)	1.7 (1.3-2.1)	1.6 (1.5-2.1)	2.0 (1.5-2.3)	1.6 (1.4-2.5)	0.656
Monocytes count (1000/µL)	0.6 (0.5-0.8)	0.6 (0.5-0.7)	0.6 (0.4-0.7)	0.6 (0.5-0.9)	0.6 (0.5-0.6)	0.861
Basophil count (1000/µL)	0.072 (0.060-0.089)	0.069 (0.061-0.081)	0.085 (0.062-0.089)	0.073 (0.051-0.095)	0.065 (0.054-0.101)	0.877
Eosinophil count (1000/µL)	0.158 (0.094-0.293)	0.231 (0.119-0.324)	0.158 (0.061-0.245)	0.189 (0.094-0.269)	0.116 (0.089-0.193)	0.477
Hematocrit (%)	43.6 ± 4.2	42.8 ± 4.6	42.9 ± 3.2	44.0 ± 5.4	45.4 ± 2.6	0.353
NLR	2.2 (1.8-3.0)	2.3 (2.0-2.9)	2.3 (1.6-3.9)	1.8 (1.2-3.0)	2.3 (1.8-2.7)	0.50
Platelet count (1000/µL)	270 (238-311)	269 (241-343)	268 (238-296)	261 (243-339)	278 (228-302)	0.977
PLR	161 (117-194)	172 (133-221)	162 (122-198)	152 (112-161)	167 (109-194)	0.288
Mean platelet volume (fL)	7.7 (7.2-8.1)	7.6 (7.3-8.1)	7.3 (6.8-8.2)	7.9 (7.7-8.1)	7.5 (7.2-8.5)	0.605
IL-6 (pg/mL)	1.0 (0.4-2.2)	1.4 (0.5-3.6)	0.9 (0.4-1.9)	0.7 (0.4-1.9)	1.1 (0.1-1.6)	0.479
MPO activity (ng/mL)	1.2 (1.1-1.3)	1.2 (1.1-1.3)	1.2 (1.1-1.3)	1.3 (1.1-2.3)	1.2 (1.0-1.3)	0.763
MPO (ng/mL)	4.0 (3.4-5.0)	4.1 (3.3-5.3)	3.8 (3.5-4.5)	4.1 (3.4-6.9)	3.6 (3.1-5.3)	0.950
Nucleosomes (AUF)	0.113 (0.082-0.158)	0.095 (0.076-0.139)	0.120 (0.082-0.183)	0.118 (0.097-0.182)	0.116 (0.067-0.148)	0.453
S100A9 (pg/mL)	239 (114-330)	225 (113-247)	248 (114-367)	215 (76-279)	288 (229-376)	0.302

Abbreviations: AUF, arbitrary unit of fluorescence; MPO, myeloperoxidase; NLR, neutrophil-to-lymphocyte ratio; PLR, platelet-to-lymphocyte ratio. Data are presented as mean±SD or median (IQR).

[a] indicates *P*<0.05 vs. stable; [b] *P*<0.05 vs. UA; [c] *P*<0.05 vs. NSTEMI; [d] *P*<0.05 vs. STEMI

Table S4. Hematological, inflammatory and neutrophil markers according to previous MI

	Previous MI	No previous MI	*P*
Neutrophil count (1000/μL)	3.99 (2.35-5.83)	2.98 (1.76-3.95)	0.163
Lymphocyte count (1000/μL)	1.69 ± 0.69	1.35 ± 0.50	0.106
Monocytes count (1000/μL)	0.47 ± 0.20	0.46 ± 0.18	0.058
Basophil count (1000/μL)	0.07 (0.03-0.11)	0.05 (0.04-0.08)	0.379
Eosinophil count (1000/μL)	0.11 (0.07-0.45)	0.12 (0.05-0.23)	0.577
Hematocrit (%)	42.10 (41.00-50.50)	42.55 (40.83-46.70)	0.612
NLR	2.29 (1.47-3.97)	2.25 (1.90-3.07)	0.859
Platelet count (1000/μL)	235 ± 73	263 ± 70	0.273
PLR	140 (105-176)	175 (149-307)	0.011
Mean platelet volume (fL)	8.16 (7.65-8.56)	7.69 (7.29-8.00)	0.058
IL-6 (pg/mL)	3.06 (0.82-4.25)	1.21 (0.12-2.95)	0.118
MPO activity (ng/mL)	1.54 (1.18-5.48)	1.50 (1.28-2.99)	0.663
MPO (ng/mL)	4.38 (2.83-9.15)	4.05 (2.85-7.23)	0.672
Nucleosomes (AUF)	0.03 (0.02-0.12)	0.04 (0.02-0.13)	0.678
S100A9 (pg/mL)	218 (144-457)	207 (131-399)	0.859
HDN SSC	66934 (63603-74387)	68082 (60814-77158)	0.669
HDN FSC	105486 ± 17757	101507 ± 16578	0.117
HDN CD11b (MFI)	13288 ± 4025	11609 ± 4032	0.081
HDN CD10 (MFI)	12316 ± 2806	12228 ± 3790	0.055
HDN CD16 (MFI)	101019 (79939-159982)	92142 (82957-133654)	0.641
HDN band cells (%)	0.02 (0.02-0.07)	0.03 (0.01-0.11)	0.648
LDN (% in PBMC)	0.90 (0.40-3.78)	0.85 (0.32-1.68)	0.574
LDN band cells (%)	2.48 (0.71-4.26)	2.81 (1.29-8.00)	0.447

Abbreviations: HDN, normal density neutrophil; LDN, low density neutrophil; MFI, median intensity of fluorescence; MPO, myeloperoxidase; NLR, neutrophil-to-lymphocyte ratio; PBMC, peripheral blood mononuclear cells; PLR, platelet-to-lymphocyte ratio; SSC, side scatter; FSC, forward scatter. Data are presented as mean±SD or median (IQR).
